# Investigation on Improving the Hot Corrosion Resistance of Selective Laser Melting Manufactured Inconel 625 by Pre-Oxidation Heat Treatment

**DOI:** 10.3390/ma18051111

**Published:** 2025-02-28

**Authors:** Teodor Adrian Badea, Mihaela Raluca Condruz

**Affiliations:** Romanian Research and Development Institute for Gas Turbines COMOTI, 220D Iuliu Maniu Av., 061126 Bucharest, Romania; raluca.condruz@comoti.ro

**Keywords:** hot corrosion, Inconel 625, oxidation, SLM, heat treatment

## Abstract

The present study was focused on assessing the molten salt-induced hot corrosion resistance of selective laser melting (SLM) manufactured Inconel 625 at 900 °C for 96 h and investigating the possibility of improving the superalloy’s corrosion resistance by applying a pre-oxidation heat treatment. The material’s hot corrosion properties were assessed in a heat-treated state (heat treatments performed at 1000 °C/1 h and 1150 °C/1 h, respectively) with and without pre-oxidation. The heat treatment at 1000 °C promoted the columnar dendrite morphology evolution, while the heat treatment at 1150 °C promoted the equiaxed dendrite morphology evolution. At 1150 °C, microstructural features specific to conventional manufactured material developed (annealing twin boundaries). They are considered a sign of anisotropy reduction due to equiaxed grains forming and it is believed that the internal stress in the material is reduced. High-temperature pre-oxidation heat treatment at 900 °C for 96 h ensured the formation of protective oxide scales with a reduced thickness (1.74 μm in the case of samples’ heat-treated at 1000 °C, and 2.22 μm in the case of samples’ heat-treated at 1150 °C, respectively). Experimentally, based on weight gain and oxide scale analysis, it was proven that pre-oxidation can improve the hot corrosion resistance of SLM manufactured Inconel 625 by forming a stable and protective oxide scale on the surface of the alloy before exposure to molten salts. The preformed oxide layer acts as a barrier for the corrosive species, reducing the formation of detrimental compounds, especially Mo-rich sulfides. Based on the tests, an improvement in corrosion resistance of up to 33.94% was observed in samples heat-treated at 1150 °C with pre-oxidation compared to samples heat-treated at 1000 °C without pre-oxidation.

## 1. Introduction

Superalloys are a category of high-performance materials developed in the late 1940s, as a solution to replace steels in applications where the operating temperatures are higher than 650 °C, like industrial and aerospace gas turbines, rocket engines, etc. This category includes alloys that are nickel/cobalt/iron-nickel-rich and exhibit structural and dimensional stability at high temperatures in harsh environments, high mechanical strength, creep and fatigue resistance, and resistance to oxidation and corrosion [1,2,3]. Many superalloys have been developed since, but there is no systematic classification system, they are classified according to the manufacturing method into cast and wrought superalloys. Additionally, cast superalloys have been classified according to their microstructure obtained after solidification (equiaxed, columnar, and single crystal), while wrought alloys have been classified according to chemical composition and by the hardening method of the nickel matrix (solid solution hardening or precipitation hardening) [4]. Recently, a third category has been added, additive manufactured superalloys [5].

Research in the field of superalloys is focused mainly on developing their properties to increase their performance [1] and extraordinary technical advances have been made in terms of chemical composition and properties [6]. Research has found that superalloys designed to ensure the best mechanical properties do not ensure maximum oxidation and corrosion resistance, and vice versa [7]. A class of superalloys that possess outstanding properties is the Inconel class developed in the 1950s, with Inconel 625 prior to Inconel 718 [8]. Although these superalloys were developed more than 70 years ago, they are still used due to their remarkable properties. The Inconel class exhibits very good properties in corrosive environments, being resistant to oxidation and corrosion at high temperatures (700–900 °C), and having very good mechanical strength in this temperature range. Both Inconel 625 and Inconel 718 are alloys characterized by a high Cr content (20–23 wt.% and 17–21 wt.%, respectively), which provides corrosion resistance by forming an oxide layer, Cr_2_O_3_, while the other alloying elements provide solid solution hardening (e.g., Mo) or precipitation hardening (e.g., Nb, Al, Ti) [4,9]. Precipitation-hardened Ni-base alloys contain Ti, Al, and/or Nb, which form hardening precipitates with nickel after appropriate heat treatment. The most common precipitates formed are γ′ (Ni_3_Al, Ni_3_Ti, Ni_3_(Ti,Al)) and γ″ (Ni_3_Nb) [4]. Although Inconel 625 has a high Nb content, it is not a precipitation-hardened superalloy, its mechanical strength is ensured by the Ni-Cr matrix hardening with Mo and Nb.

The most representative components manufactured from Ni-based superalloys that operate in harsh environments, as in gas turbine engines (including micro turboprops for multirole unmanned aircraft), are turbine blades. The environment in which turbine blades operate has a major influence on their durability. Gas turbine engines capture a significant amount of air during operation, along with impurities, leading to the degradation of turbine components either due to erosion or impact [10]. Hard particles of sand, salt, and dust can reach the combustion chamber, where deposits can form and detach during operation, reaching the turbine blades and causing erosion [11]. Other sources that can lead to blade erosion are the coke deposits formed at the injection nozzle where the fuel is introduced into the combustion chamber or the thermal barrier ceramic coatings (TBCs) that can degrade, exfoliate due to thermal shock, and can reach the turbine blades or other components [10]. In addition to erosion, turbine blades can also be degraded by corrosion. In this case, corrosion can be achieved through the specific mechanisms of sulfidation and hot corrosion. Hot corrosion is a form of accelerated corrosion associated with contamination with a series of alkali metals such as Na and K that react with the S contained in fuel. Sulfidation is considered a form of hot corrosion in which residues containing alkali sulfates are deposited on the turbine blades over time, leading to corrosion [12]. Depending on the conditions under which hot corrosion occurs, it can be type I corrosion (high-temperature hot corrosion) or type II corrosion (low-temperature hot corrosion) [12]. Type I corrosion was identified in the 1950s for gas turbine engines in the aviation sector as occurring in the temperature range 800–927 °C and in the presence of Na_2_SO_4_ resulting from fuel combustion [12,13]. Type II corrosion was identified in the mid-1970s in the case of gas turbine engines from the naval sector, being a form of rapid oxidation that occurs in the temperature range 600–760 °C in the presence of SO_2_ [12,13] and is characterized by a pitting attack. Given that sulfur is a natural impurity and that various compounds result from fuel combustion, measures have been taken to limit the sulfur content in the fuel to reduce the risk of hot corrosion of turbine engine components [13].

Several studies were conducted regarding the hot corrosion of Inconel 625 alloys. For example, in the study of M.K. Hariharan et al. [14], the molten salt-induced hot corrosion behavior of coated and uncoated Incoloy 800H and Inconel 625 was assessed at 1000 °C for 8 h, 12 h, and 16 h. They observed that the coated specimens present a higher resistance to hot corrosion than uncoated specimens. Li et al. [15] researched the corrosion behavior of hot extruded Inconel 625 in a molten salt system (Na_2_SO_4_ and V_2_O_5_) at different temperatures and durations. Their study highlights that the alloy’s damage increases with time at a constant temperature. First, a dense oxide layer (Cr_2_O_3_ and NiO) developed, and then, the exposure to molten salts for 24 h promoted a chemical reaction between the alloy and V resulting in the formation of CrVO_4_ and Ni_3_V_2_O_8_. S passed through the oxide film and entered the matrix, leading to the formation of internal sulfides, including Ni-, Cr-, and Mo-based sulfides, which accelerated the intergranular and intracrystalline corrosion.

Khorsand et al. [16] assessed the corrosion resistance of Inconel 625 in a molten nitrate salt (40 wt.% KNO_3_–60 wt.% NaNO_3_) at 500 °C and 600 °C. The resulting curves showed that with the increase in temperature, the oxidation rate and mass gain increased, following a near-parabolic law. The corrosion results confirm that during the exposure of Inconel 625 to the molten salts, nickel dissolves because of the non-protective NiO layer formed. The formation of a non-protective oxide layer resulted in low corrosion resistance. Y.J. Ma et al. [17] investigated the corrosion behavior of Inconel 625 at 800 °C and 900 °C in molten salts (75 wt.% Na_2_SO_4_ + 25 wt.% K_2_SO_4_). The corrosion mechanism of Inconel 625 alloy after hot corrosion was mainly an alkaline melting mechanism. The formed Cr_2_O_3_ oxide scale dissolved in molten salts and formed Na_2_CrO_4_, which did not ensure the alloy’s protection. Further, the corrosion promoted the formation of a discontinuous oxide layer at the alloy-corrosion layer interface instead of Cr_2_O_3_, as the region was depleted in Cr and it allowed O and S to infiltrate and corrode the matrix. At 800 °C, the surface layer formed consisted of flaky Cr_2_O_3_ and spinel-like NiCr_2_O_4_, while at 900 °C, defects appeared in the scale formed at the alloy surface, the oxide scale consisted of three layers: the outer layer was composed of NiCr_2_O_4_ and NiO, the middle layer was a dense Cr_2_O_3_, and the inner layer was composed of sulfides (Cr_2_S_3_ and Ni_3_S_2_), oxides (Cr_2_O_3_ and NiO), telluride, etc.

Jiang et al. [18] studied the effect of thermal-induced phase transformation on the corrosion behavior of Inconel 625 alloy by static corrosion test with NaCl-KCl-MgCl_2_ molten salt at 700 °C for 400 h. They observed that a selective dissolution of Cr and infiltration of Mg and O through the diffusion channels are the main processes of corrosion. The surface corrosion products were composed of MgO/MgCr_2_O_4_ and subsurface corrosion holes formed in Inconel 625 alloy.

Hongfei et al. [19] researched corrosion in molten salts (47 wt.% Na_2_SO_4_ + 53 wt.% MgSO_4_) of the laser-clad manufactured Inconel 625 at 900 °C for 12 h. They observed that the corrosion process starts with the formation of Cr oxides on material surface, which, in contact with the molten salts, promotes the outward diffusion of Cr atoms and accelerates the precipitation of δ-Ni_3_(Nb, Mo), favoring the degradation of the material through spallation. They stated that it is very important to study the influence of oxidation preceding the corrosion process and the amount of δ phase in the superalloy.

The goal of the present study was to assess the hot corrosion behavior of selective laser melting (SLM) manufactured Inconel 625 at high temperatures and to investigate the possibility of improving the superalloy hot corrosion resistance by applying a pre-oxidation heat treatment. This type of study was also performed by various authors in the case of other alloys, and they observed a significant improvement in the corrosion resistance of the baseline material. For example, a study was reported regarding the improvement of FeCrAl alloy’s corrosion resistance at high temperatures in different conditions by pre-oxidation. In this case, the pre-oxidation generated a protective alumina oxide, and the corrosion resistance of the alloy was improved [20]. The effect of pre-oxidation treatment on corrosion resistance of FeCoSiBPC amorphous alloy was also investigated by Zhang et al. [21]. The oxide layer formed by the pre-oxidation treatment had a protective effect and promoted the formation of a dense passivation film.

Lehmusto et al. [22] studied pre-oxidation to increase corrosion resistance of commercial superheater steels (10CrMo9-10, AISI 347, and Sanicro 28). Samples were pre-oxidized under various conditions and then exposed to potassium chloride for 168 h at 550 °C under dry conditions. In the case of 10CrMo9-10 and AISI 347, despite differences in oxide scale composition and structure being registered, their corrosion resistance could not be improved by any of the studied pre-oxidation procedures. In contrast, pre-oxidation conditions resulting in chromium-enriched oxides improved the corrosion resistance of Sanicro 28 steel.

Yang et al. [23] investigated the hot corrosion behavior of the single-crystal Ni-based superalloy N5 in molten salts (Na_2_SO_4_ + K_2_SO_4_) and the influence of pre-oxidation treatments conducted at 900 °C, 1000 °C, and 1100 °C for 20 h and 100 h. The oxide scale formed had a multilayered structure and different percentages of Ta oxides. Among all the samples, those pre-oxidized at 900 °C exhibited the highest corrosion resistance. A porous structure with many internal oxides and sulfides was observed when the alloy was pre-oxidized at 1000 °C and 1100 °C. The alloy without pre-oxidation directly reacted with the molten salts and formed a porous layer consisting of oxides and sulfides.

Wo et al. [24] studied the isothermal oxidation of a new polycrystalline Ni-based superalloy—C19—at 800 °C for 1000 h and its modification with pre-oxidation. The results were compared with the results obtained for Nimonic 105 alloy. The pre-oxidation treatment at 1100 °C for 1 h was applied and was shown to dramatically improve oxidation resistance during subsequent exposure at 800 °C.

Chen et al. [25] studied the effects of different surface pre-oxides on the hot corrosion properties of three nickel-based single-crystal superalloys with different Cr compositions at 900 °C in molten salts (75 wt.% Na_2_SO_4_ + 25 wt.% NaCl). They concluded that the samples with 20 wt.% Cr pre-oxidized developed a Cr_2_O_3_ on the surface, while the samples containing less than 10 wt.% Cr after pre-oxidation formed an oxide scale composed of NiO and Al_2_O_3_. Cr_2_O_3_ has a strong hot corrosion stability, but it is difficult to maintain a large oxide thickness by pre-oxidation due to its high volatility, so the effect of pre-oxidation was not obvious. NiO was easily consumed during hot corrosion in molten salts but promoted the formation of a dense beneficial Al_2_O_3_ layer during its consumption, preventing the reaction between the alloy and the molten salt. Sulfides formed during hot corrosion, especially Cr-S compounds. They concluded that the formation of Mo-S compounds in a large amount means that the hot corrosion is carried out to a catastrophic state.

Based on the literature review, it can be stated that there are significant results that prove the possibility of improving the hot corrosion resistance of alloys by pre-oxidation heat treatment. Therefore, it was considered appropriate to deepen this research in the case of an additive manufactured Ni-based superalloy, Inconel 625. The originality of this research is demonstrated by the comprehensive analysis of the additive manufactured Inconel 625 in various states and the subsequent evaluation of how these states affect the material’s corrosion resistance, as such a study was not identified in the literature. The novelty of this research lies in various aspects that were investigated, for example, heat treatment temperature impact on microstructural features of additive manufactured Inconel 625 (microstructure that differs from that of a conventional manufactured material) in the context of oxidation and hot corrosion resistance, the role of the oxide scale formed during pre-oxidation against the corrosive molten salts, and the behavior of the material in molten salt environments at high temperatures in oxidized and unoxidized conditions. The investigation was performed sequentially, focusing on both oxidation and hot corrosion conditions, not individually. The results obtained can be used as input for designing and manufacturing high-temperature components that operate in aggressive environments.

## 2. Materials and Methods

The research was conducted on Inconel 625 samples additive manufactured by SLM. Inconel 625 powder manufactured by LPW Technology Ltd. (Runcon, UK subsidiary of Carpenter Additive) was used as feedstock, and it was characterized by a powder size in the range of 15–45 μm and a chemical composition that is presented in Table 1.

The manufacturing process was performed on a Lasertec 30 SLM machine (DMG Mori Romania, Pitesti, Romania), using the following process parameters: 250 W laser power, 750 mm/s scanning speed, 50 μm layer thickness, 0.11 mm hatch distance, and a scanning strategy at 90°. The process was performed on an Inconel 625 building plate preheated at 80 °C, in an argon atmosphere. Samples of 20 mm × 15 mm × 4 mm were cut from SLM manufactured plates and were polished on SiC abrasive paper up to 1200 grit granulation. Next, they were cleaned with water, ultrasonicated in acetone, and dried in air on filter paper. Dried samples were heat-treated at two different temperatures, 1000 °C (hereafter called HT1000) and 1150 °C (HT1150). HT1000 consisted of heating from room temperature up to 1000 °C, maintaining for 1 h at 1000 °C, followed by air cooling. HT1150 consisted of heating from room temperature up to 1150 °C, maintaining for 1 h at 1150 °C, followed by air cooling. For each heat treatment (HT1000 and HT1150), 7 samples were used as follows: 1 sample was used for microstructural analysis in a heat-treated condition; 3 heat-treated samples were further subjected to hot corrosion heat treatment; and 3 heat-treated samples were pre-oxidized at 900 °C for 96 h and afterwards were subjected to the hot corrosion heat treatment with molten salts. After the HT1000 and HT1150 heat treatments were performed, all samples were polished and cleaned again to remove the superficial oxide layer. Alumina crucibles were also cleaned with acetone. The samples were weighted with and without the crucibles using a PX224 Pioneer (Ohaus Europe GmbH, Nänikon, Switzerland) analytical balance (accuracy 10^−4^ g). The pre-oxidation heat treatment was performed, which consisted of heating from room temperature until 900 °C was reached, maintained at this temperature for 96 h, and afterwards they were cooled in the furnace. The hot corrosion tests were performed on oxidized and unoxidized samples using a mixture of 50 wt.% Na_2_SO_4_ and 50 wt.% V_2_O_5_ powders, which were spread on the samples’ polished surface with a brush. The Na_2_SO_4_ and V_2_O_5_ powders with purities of 99% and 99.6%, respectively, were acquired from Carlo Erba Reagents SAS (Val-de-Reuil, France). The area density of the mixture was approximative 5 mg/cm^2^ in the case of each sample. The hot corrosion treatment was performed by heating from room temperature to 900 °C, holding at temperature of 900 °C for 8 h, 48 h, and 96 h, respectively, followed by cooling in the furnace. For all heat treatments performed during the present research, a Nabertherm LH 30/14 furnace (Nabertherm GmbH, Lilienthal, Germany) was used. All samples were weighed to assess their weight gain after each stage of heat treatment was performed.

The HT1000 and HT1150 samples were metallographically prepared by grinding and polishing, and were etched by immersion in Aqua Regia etchant for 10–15 s to highlight the microstructural features of the material in a heat-treated state. Optical microscopy was performed on an Axio Vert. A1 MAT Optical microscope (Carl Zeiss Microscopy GmbH, Jena, Germany).

**Figure 1:**
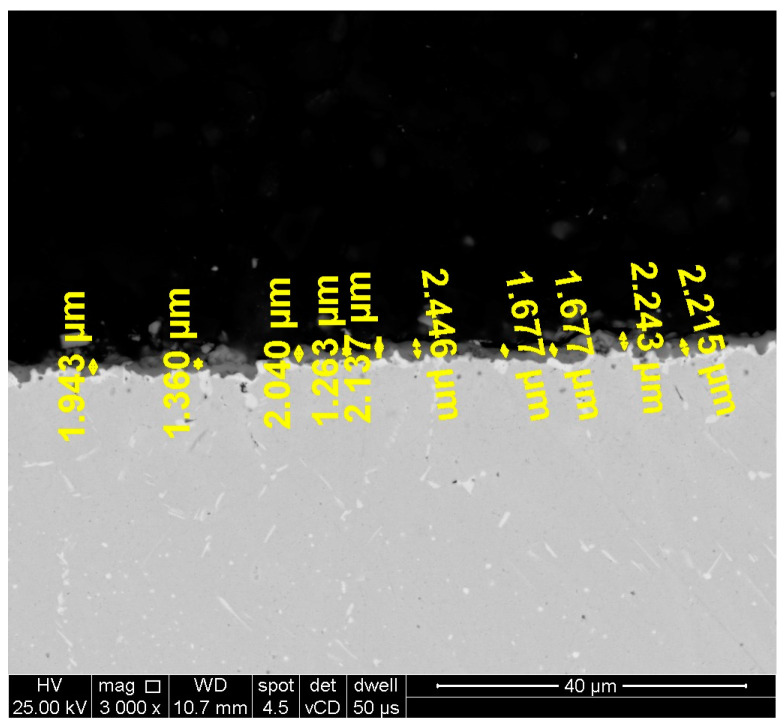
Example of how the thickness measurements were made in one micro-area.

Microstructural investigations on the oxidized and unoxidized samples after hot corrosion were performed by scanning electron microscopy (SEM) to assess the thickness of the layer formed on the surface. The layer thickness analysis was performed on SEM images (3000× magnification) obtained of the cross section of one sample of each category (heat-treated samples at 1000 °C/1150 °C oxidized, heat-treated samples at 1000 °C/1150 °C unoxidized after hot corrosion, and heat-treated samples at 1000 °C/1150 °C oxidized after hot corrosion). For each sample, 10-layer thickness measurements were performed in 2 micro-areas and the average layer thickness value was reported for each type of sample. An example of how the thickness measurements were made in one micro-area can be observed in Figure 1.

For SEM analysis, the F50 Inspect SEM equipped with an energy-dispersive X-ray spectrometer (EDS) EDAX APEX 2i, SDD Apollo X detector (FEI Company, Brno, Czech Republic) and EDAX Genesis software v6.29 (EDAX Inc. Ametek MAD, Mahwah, NJ, USA) were used. Backscattered electron images with a low-voltage high-contrast detector (vCD detector, FEI Company) were captured to highlight the chemical composition differences at the interface between the layer formed and the substrate. A microcompositional qualitative analysis on 2 micro-areas from the layer formed on the surface of oxidized and unoxidized corroded samples was performed by SEM-EDS on the vCD images obtained of the cross-section of the samples, using an acceleration voltage of 25 kV, a spot size of 4.5 nm, and a working distance between 10.7 and 11.2 mm.

## 3. Results and Discussions

**Figure 2:**
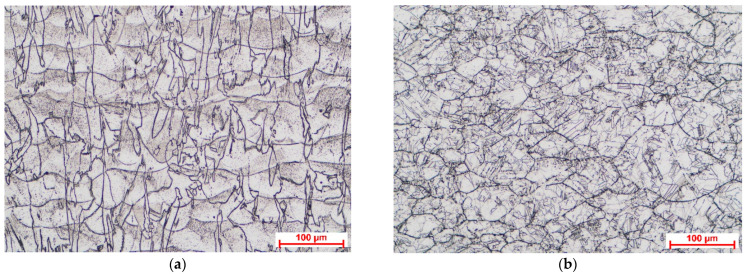
The microstructure of SLM Inconel 625 after HT1000 (**a**) and after the HT1150 (**b**).

Figure 2 shows the optical microscopy images taken to highlight the microstructural features of the material in the two different heat-treated conditions, after the heat treatment at 1000 °C for 1 h and after the heat treatment at 1150 °C for 1 h. The images were taken in the ZX plane of the samples (on the SLM process building direction, parallel to the Z-axis).

The high-temperature heat treatments ensured the recrystallization of the material. The heat treatment at 1000 °C promoted columnar dendrite morphology evolution characterized by columnar grains distributed over multiple layers parallel to the building direction, while the heat treatment at 1150 °C promoted equiaxed dendrite morphology evolution. In the case of the sample heat-treated at the lower temperature, the layer boundaries were still visible, while the high temperature promoted the disappearance of layer boundaries, a conclusion also supported by the study performed by Hu et al. [26]. Annealing twin boundaries were observed in the case of the sample heat-treated at 1150 °C, which can be considered a sign of release of the residual stress stored during additive manufacturing process, as was presented by Li et al. [27]. Full recrystallization of the microstructure of additive manufactured Inconel 625 after heat treatment at 1100 °C for 3 h was observed by De Terris et al. [28], and they also concluded that this full recrystallization can eliminate the material’s anisotropy. The microstructural features obtained after the heat treatment at 1150 °C were similar with those obtained by Nguejio et al. [29] in the case of SLM manufactured Inconel 625 annealed for 1 h at 1100 °C and conventional manufactured (wrought) Inconel 625 annealed for 1 h at various temperatures in range of 700–1100 °C.

Regarding phase development, the existing literature presents that in an as-printed state, SLM manufactured Inconel 625 consists in the γ matrix [27,30,31], fine γ″ (N_3_Nb) platelets being identified in case of EBM (electron beam melting) manufactured Inconel 625 [32]. In heat-treated conditions, various intermetallic phases can precipitate (δ—Ni_3_Nb, Laves—NbCr_2_, Nb-/Mo-rich carbides) [31,33]. Li C. et al. [27] heat-treated Inconel 625 at 870 °C, 980 °C, and 1150 °C. At 870 °C, the microstructure was similar to the microstructure observed in the as-printed material; the melt pool boundaries were slightly blurred compared to those from the as-printed material. At 980 °C, the material showed signs of recrystallization, while complete recrystallization was identified at 1150 °C. They concluded that the precipitation of the δ phase was possible after the 870 °C heat treatment, and that heat treatment at higher temperatures promoted the dissolution of the δ phase and the precipitation of carbides at grain boundaries, which were observed in the samples heat-treated at 1150 °C. The precipitation of carbides was also observed for SLM manufactured Inconel 625 heat-treated at 1000 °C and 1150 °C by Li S. et al. [31]. Keller et al. [30] also reported the presence of Laves phase after heat treatment at 1150 °C of SLM manufactured Inconel 625 along with carbides. In another study, it was reported that Laves and carbides are common phases encountered in conventionally manufactured Inconel 625 alloy, with intermediate C/Nb ratios leading firstly to γ and NbC development followed by Laves at the end of solidification [33].

The microstructure of additive manufactured Inconel 625 has a significant influence on the corrosion resistance of the material. Residual stress, some precipitates, and grain dimensions can affect corrosion resistance, but applying a proper heat treatment could be beneficial for improving corrosion resistance.

**Figure 3:**
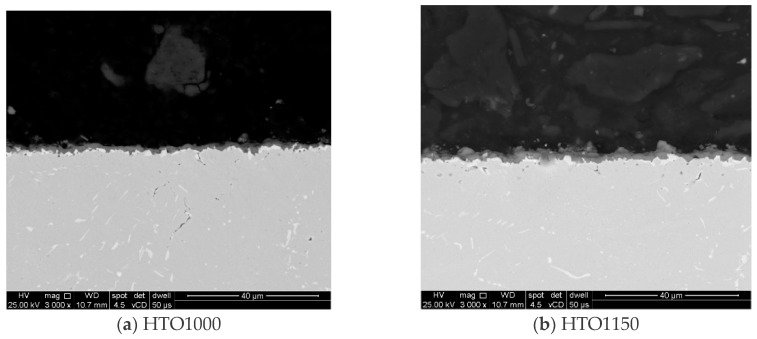
SEM images with the heat-treated samples at 1000 °C and 1150 °C that were oxidized at 900 °C for 96 h.

The pre-oxidation heat treatment was performed on HT1000 and HT1150 samples and an analysis was performed to measure the thickness of the layer formed on Inconel 625 samples’ surface after oxidation. Figure 3 shows representative SEM images made in the cross-section of the heat-treated samples that were oxidized at 900 °C for 96 h. The oxide layer thickness measurements made on SEM images revealed that the samples oxidized at 1000 °C presented a lower oxide layer thickness compared to samples oxidized at 1150 °C (1.74 μm with a standard deviation of 0.38 µm, and 2.22 μm with a standard deviation of 0.36 µm, respectively). The higher heat treatment temperature changes the thermodynamics of oxidation and ensures an increased mobility of metal atoms and oxygen ions, enhancing the kinetics of oxide layer growth.

**Figure 4:**
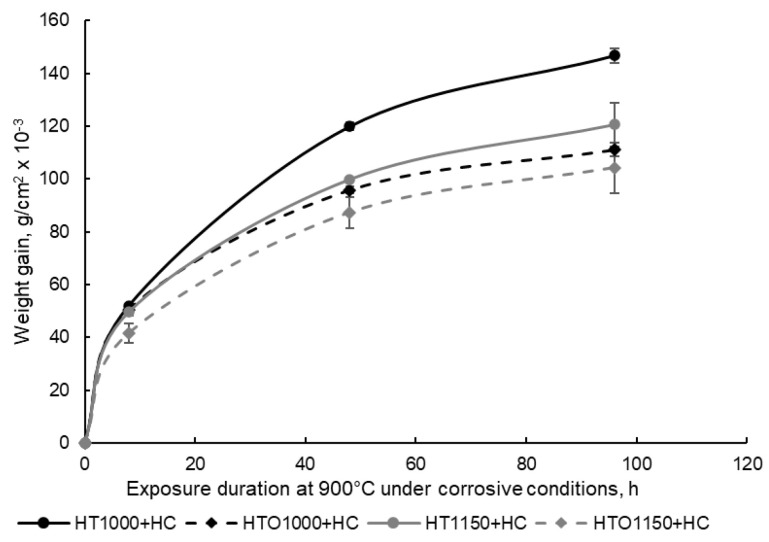
Kinetic curves of the hot corroded samples at 900 °C for 96 h (HT—samples in heat-treated condition without oxidation, HTO—samples in heat-treated condition with oxidation at 900 °C for 96 h, HC—hot corrosion).

To assess the influence of the high-temperature heat treatment and pre-oxidation on the corrosion resistance of the material, a weight gain analysis was performed. The samples were weighed in oxidized and unoxidized conditions after hot corrosion in molten salts. In Figure 4, the average weight gain of the samples is presented after hot corrosion.

Based on the graphic representation from Figure 4, it was determined that the samples’ weight gain increased simultaneously with the increase in the hot corrosion duration. Nevertheless, it was observed that the samples that were pre-oxidized had a lower weight gain (regardless of the initial heat treatment temperature applied). The process of hot corrosion in molten salts at 900 °C for 96 h followed a parabolic low characterized by calculated kp values of 6.23 × 10^−8^ g^2^·cm^4^·s^−1^ for the HT1000 + HC samples; 3.57 × 10^−8^ g^2^·cm^4^·s^−1^ for the HTO1000 + HC sample; 4.21 × 10^−8^ g^2^·cm^4^·s^−1^ for the HT1150 + HC samples; and 3.14 × 10^−8^ g^2^·cm^4^·s^−1^ for the HTO1150 + HC samples. The kp values show how fast the layer formed at the samples’ surface grows (the higher the kp value, the faster the surface layer growth, and the lower the kp, the slower layer formation). The lowest kp value was recorded for the samples of HTO1150 + HC, followed by the samples of HTO1000 + HC and HT1150 + HC, and the highest value being calculated for the samples of HT1000 + HC. Material with a lower kp has better corrosion resistance since the protective oxide layer formed grows more slowly and efficiently.

Zhao et al. [34], who studied the corrosion performance of welding-based additive manufactured (WAAM) Inconel 625 (determined by potentiodynamic polarization tests) heat-treated at 980 °C/1 h and at 1100 °C/1 h, observed the dissolution of detrimental phases (i.e., δ and Laves) at higher temperatures and the presence of a large number of stable twin grain boundaries resulting in improved corrosion resistance of the material. The twin grain boundaries were also observed in the present study in the case of the Inconel 625 heat-treated at 1150 °C.

**Figure 5:**
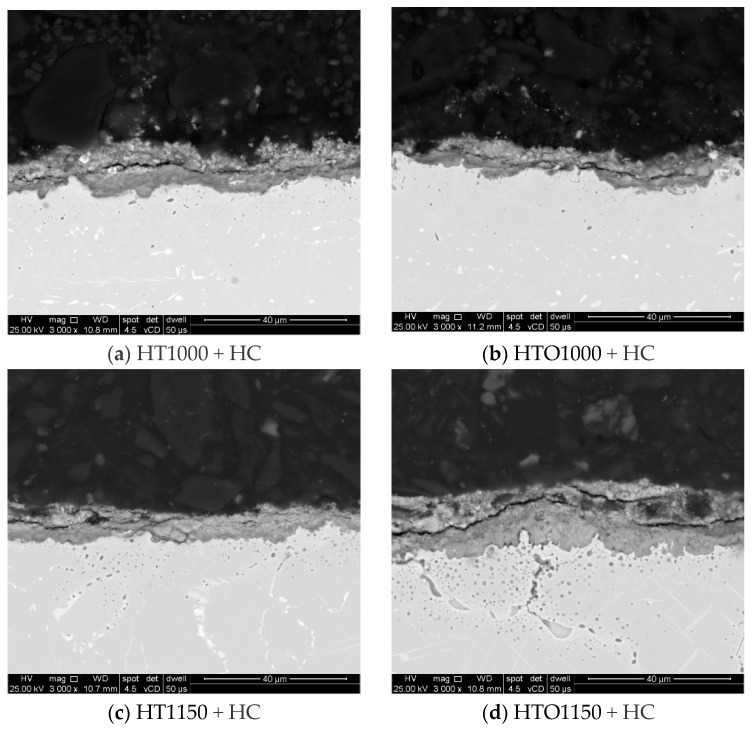
SEM images with the oxidized and unoxidized hot corroded samples’ cross-section.

During the weight gain analysis, it was observed that in the case of the HT1000 + HC and HT1150 + HC samples, a spallation of the scale occurred, which was found in the crucible. This was also observed on the SEM images, which are presented in Figure 5.

**Figure 6:**
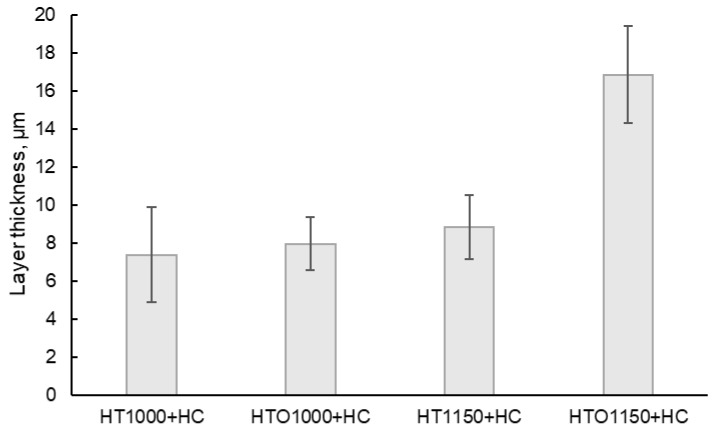
Layer thickness developed on the surface of the samples.

Measurement of the layer thickness performed on SEM images also supported the occurrence of spallation (Figure 6), as the oxidized samples present a higher layer thickness than the unoxidized samples, even if they had a better corrosion resistance (based on the calculated kp value). The salts interact with the superalloy, forming a layer that exfoliates cyclically until the entire amount of salt is neutralized by the superalloy. A large layer that has not yet been exfoliated may indicate that the material has managed to neutralize a larger amount of salt in a short period. The higher the oxide layer thickness formed during the pre-oxidation heat treatment, the higher the possibility to ensure a better protection of the baseline material as the molten salts interact with this oxide layer rather than with the material.

These results are correlated with the recorded kp values (the highest kp value being recorded for the HT1000 + HC samples, characterized by the most aggressive corrosion, and the lowest kp value being recorded for the HTO1150 + HC sample, characterized by the most gentile corrosive process) and with the weight gain analysis (the higher weight gain trendline being recorded for the HT1000 + HC samples, which were those that were corroded the most).

From the microstructure perspective (ensured by the heat treatment temperature applied), it was concluded that the best performance was ensured by the microstructure obtained in the case of heat-treated samples at 1150 °C. This conclusion can be justified by the presence of the specific microstructural features obtained in the case of the higher-temperature heat treatment as it promoted the disappearance of layer boundaries and the presence of annealing twin boundaries (considered a sign of residual stress release, stress that was stored during SLM manufacturing). High-temperature heat treatments above 1000 °C can reverse sensitization by restoring chromium to the grain boundaries, improving overall corrosion resistance. This results in more homogeneous protection across the alloy’s surface, especially in additive manufactured materials, where microstructural anisotropy can make various regions more prone to corrosion.

According to previous studies performed by the authors regarding the high temperature oxidation resistance of SLM manufactured Inconel 625 [35], and hot corrosion behavior of Inconel 625 [36], it was determined that the oxidation process promotes the formation of Cr_2_O_3_ and NiCr_2_O_4_ spinel. The presence of molten Na_2_SO_4_ and V_2_O_5_ disrupts the stability of this oxide layer and ensures the material’s corrosion. Furthermore, the high-temperature pre-oxidation treatment ensured a higher material resistance in the presence of molten salts due to the development of the protective scale. A more severe corrosive effect was registered for the unoxidized samples.

**Figure 7:**
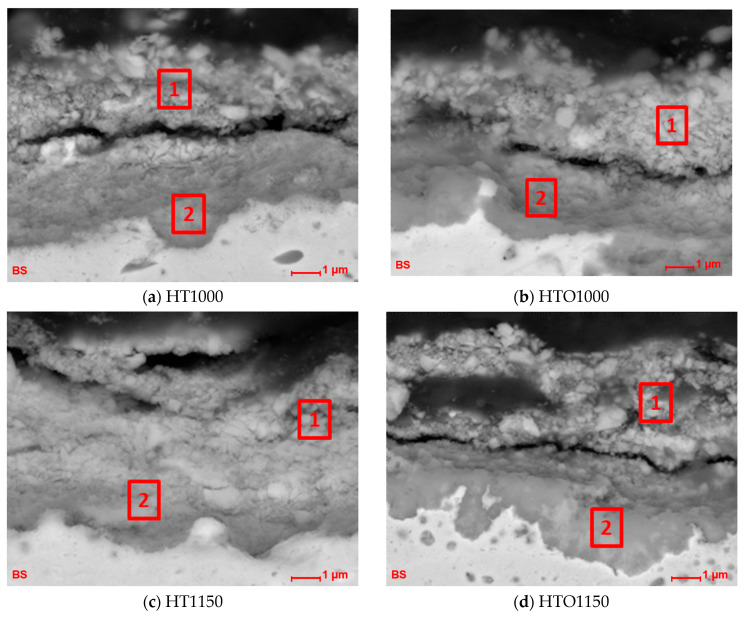
The micro-areas where SEM-EDS analysis was performed in the layer developed on the samples’ surfaces (with 1 and 2 are marked the areas where the chemical composition was determined).

A SEM-EDS qualitative chemical analysis was performed to identify the chemical elements from various micro-areas from the scale formed in the case of oxidized and unoxidized hot corroded samples. Representative SEM images of the areas where the chemical analysis was performed are presented in Figure 7 and the chemical compositions obtained are presented in Table 2.

Based on the presented chemical compositions obtained by SEM-EDS in specific areas, it can be stated that the oxidation process promoted the formation of Cr_2_O_3_; the higher the temperature of the heat treatment applied, the higher the Cr and O contents. The presence of Ni is a sign of the NiCr_2_O_4_ development.

Staszewska et al. [37] studied the mechanism and kinetics of oxidation of the superalloys Inconel 617 and Inconel 625. For these materials, the authors observed that after prolonged oxidation, a compact oxide layer was formed on the surface of the material, consisting of an outer layer of Cr_2_O_3_ and an inner layer composed of a spinel NiCr_2_O_4_, together with other types of oxides. The spinel NiCr_2_O_4_ was also observed by Vesel et al. [38] for Inconel 625, while a similar spinel (NiAl_2_O_4_) was identified by Zhang et al. [39].

High concentrations of chromium and molybdenum, as well as the presence of niobium, which cause the formation of a Cr_2_O_3_ protective film together with NbCrO_4_ and, in some cases, a Cr-Mo- and Nb-rich layer, are the most important compositional and microstructural features of alloy Inconel 625 [40].

Na_2_SO_4_ and V_2_O_5_ react at high temperatures and form a highly corrosive eutectic mixture known generically as molten salts that attack metal surfaces, causing material’s hot corrosion. The molten V_2_O_5_ acts as an oxidizing agent, further accelerating the oxidation of the base material. The presence of sulfur from Na_2_SO_4_ contributes to sulfidation, where sulfur combines with metal atoms to form sulfides, which are less protective than oxides and lead to rapid material degradation [15]. In the case of pre-oxidized material, the protective oxide layer formed during pre-oxidation heat treatment inhibits the diffusion of V and S from the molten salts to the baseline material. The protective Cr_2_O_3_ can react with V_2_O_5_ and the stable CrVO_4_ can form, preventing the penetration of V into the substrate [41].

As was expected, in the second area of the oxidized and corroded samples, a higher oxygen content was observed compared to the samples that were just corroded without pre-oxidation. A higher oxygen content was observed also on the samples heat-treated at a higher temperature than those heat-treated at a lower temperature, the oxidation process being more intense at high temperatures.

Furthermore, it is known that the infiltration of S into the matrix leads to the formation of internal sulfides, including Ni-, Cr-, and Mo-based sulfides, which accelerate intergranular and intracrystalline corrosion [15]. The S content in this study was lower in the second micro-area compared with the first micro-area investigated (surface), and a lower S content was identified in the areas of the pre-oxidized samples compared with the unoxidized samples. No significant differences were observed in terms of the V content. In this case, the development of Cr_2_O_3_ prevented S diffusion, reducing sulfide formation at the baseline material–oxide interface. Even if spallation of the formed layer can be observed, Inconel 625 can re-oxidize Cr and Ni to regenerate a protective oxide layer over a limited period of time. The higher thickness of the oxide layer formed during pre-oxidation heat treatment slowed down the penetration of molten salts and it can annihilate them, extending the corrosion resistance of Inconel 625 and extending its service time.

A balanced Mo content (typically 8–10 wt.%, 8.90 wt.% in the present case) is critical for maintaining a good trade-off between oxidation resistance and hot corrosion resistance of Inconel 625. At high temperatures, Mo promotes Cr diffusion and contributes to the formation of protective Cr_2_O_3_. Molten salts dissolve the protective oxide layer by exposing the underlying metal to further oxidation and sulfidation, leading to severe material losses.

Nevertheless, based on the chemical compositions presented in Table 2, it can be observed that the Mo content was lower in the surface scale formed on the oxidized samples (regardless of the temperature used for heat treatment) than on the unoxidized samples. Also, the heat treatment at 1150 °C promoted a lower content in Mo-rich components in the scales formed than the heat treatment at 1000 °C. At 1000 °C, the Mo from the oxide layers had a lower volatility, and increasing the temperature up to 1150 °C increased Mo oxide volatility. However, in high-temperature hot corrosion, Mo can react with sulfur to form molybdenum sulfides, which are detrimental to the alloy’s integrity.

A high Mo content was registered in the scale formed after material’s hot corrosion in molten salts, regardless of the treatment conditions applied previously. The Mo from the matrix material can interact with the salts during hot corrosion and, due to acidic fluxes, can result in the material’s catastrophic failure [42,43]. This phenomenon was identified also in cases of hot corrosion of other type of alloys [42,44] and can cause increased spalling of the scale formed on the surface and damage of the baseline material. Mo in Inconel 625 is a beneficial alloying element for strength and corrosion resistance but becomes a liability in molten sulfate-vanadate environments. Mo forms volatile and reactive molybdate compounds that worsen hot corrosion by increasing fluxing of the protective oxide layer.

The samples where the highest Mo contents were registered were those where the highest weight gain was registered in the case of hot corrosion, which was a sign that the Mo reacts with S (a higher S content being registered for unoxidized samples).

The oxide and spinel layer were present in a higher content in micro-area two, as can be observed when all wt.% of the elements of interest, O, Cr, and Ni, are summed (Table 3).

Based on the values presented in Table 3, it can be said that, in the case of pre-oxidized samples, a higher proportion of protective oxides were developed that interact with the molten salts and diminish their effect, and the formed oxide ensured the annihilation of the corrosive salts. The highest elemental content of O, Cr, and Ni were recorded in micro-area two of the HTO1150 + HC sample (82.92 wt.%) compared with the lowest recorded value in the case of unoxidized sample HT1000 + HC (64.76 wt.%). Regarding the values recorded in micro-area one (the top surface of the scale), the highest contents of O, Cr, and Ni were also recorded in the HTO1150 + HC sample (70.01 wt.%) and the lowest recorded value in the unoxidized sample HT1000 + HC (49.85 wt.%).

The experimental analyses performed showed that high-temperature heat treatment along with a pre-oxidation heat treatment can have a beneficial effect on the SLM manufactured Inconel 625 exposed to hot corrosion in molten salts.

## 4. Conclusions

The aim of this study was to evaluate the hot corrosion behavior in molten salts (900 °C/96 h) of SLM manufactured Inconel 625, previously heat-treated at 1000 °C/1 h and 1150 °C/1 h, and to explore the potential for enhancing the superalloy’s hot corrosion resistance through a pre-oxidation heat treatment (900 °C/96 h). The conclusions of the present study are as follows:

- The heat treatments ensured the recrystallization of Inconel 625, the 1000 °C/1 h promoted the columnar dendrite morphology evolution, and the 1150 °C/1 h promoted the equiaxed dendrite morphology evolution (microstructural features specific to conventional manufactured material—annealing twin boundaries).
- Pre-oxidation heat treatment at 900 °C/96 h resulted in the formation of reduced-thickness oxide scales (1.74 μm for the samples’ heat-treated at 1000 °C/1 h, and 2.22 μm for the samples’ heat-treated at 1150 °C/1 h).
- The pre-oxidation heat treatment promotes the development of an oxide scale (consisting mainly of Cr_2_O_3_ and NiCr_2_O_4_), which acts as a barrier to corrosive species, reducing the formation of detrimental compounds, especially Mo-rich sulfides, during hot corrosion in molten salts.
- Pre-oxidation allows for the development of a more uniform and adherent oxide layer compared to an oxide layer that forms in situ during service, which can be less uniform and more prone to spalling.
- Based on the kinetic curves and the kp values, it was determined that the best hot corrosion resistance was registered for pre-oxidized Inconel 625 heat-treated at 1150 °C/1 h and the lowest hot corrosion resistance was registered for unoxidized Inconel 625 heat-treated at 1000 °C/1 h.
- It was proven experimentally that pre-oxidation heat treatment can improve the hot corrosion resistance of SLM manufactured Inconel 625 by forming a stable and protective oxide scale on the surface of the alloy before exposing it to molten salts.

Future studies will be conducted regarding the performance of different additive manufactured Ni-based superalloys in corrosive environments, studying the influence of alloying elements, anisotropy, and lifecycle analysis of gas turbine components that are in service in harsh environments.

## Figures and Tables

**Table 1:** Chemical composition of Inconel 625 powder (wt.%).

Elements	Ni	Cr	Mo	Fe	Nb	Co	Mn	Al	Ti	Si	C
Inconel 625 powder	62.26	20.70	8.90	4.10	3.77	0.10	0.01	0.06	0.07	0.01	0.02

**Table 2:** Chemical compositions obtained by SEM-EDS in specific areas from Figure 7.

Elements, wt.%		O	Na	Mo	S	V	Cr	Fe	Ni
HT1000 + HC	area 1	14.98	2.23	30.26	13.75	1.06	12.14	2.86	22.73
	area 2	20.88	2	21.16	9.14	0.9	17.68	2.04	26.2
HTO1000 + HC	area 1	13.38	2.47	24.25	10.96	1.13	21.43	3.17	23.2
	area 2	24.79	1.74	16.23	7.3	1.01	22.4	4.33	22.2
HT1150 + HC	area 1	19.03	1.85	23.69	10.76	1	16.8	4.14	22.73
	area 2	24.31	1.84	17.43	7.73	0.89	24.75	2.11	20.95
HTO1150 + HC	area 1	7.83	0.99	17.69	7.77	1.27	30.67	4.79	28.99
	area 2	26.95	1.97	5.72	2.36	0.92	33.22	6.11	22.75

**Table 3:** Summary of chemical composition of elements that form Cr_2_O_3_ and NiCr_2_O_4_.

Sample	HT1000 + HC		HTO1000 + HC		HT1150 + HC		HTO1150 + HC	
Area	Area 1	Area 2	Area 1	Area 2	Area 1	Area 2	Area 1	Area 2
O + Cr + Ni [wt.%]	49.85	64.76	58.01	69.39	58.56	70.01	67.49	82.92

## Data Availability

The original contributions presented in the study are included in the article, further inquiries can be directed to the corresponding author.
